# Trans-scaphoid perilunate fracture-dislocation requiring carpal tunnel release in college football players: a case report

**DOI:** 10.1097/RC9.0000000000000586

**Published:** 2026-06-25

**Authors:** Stephen Rossettie, James Anundson, Patrick Burroughs, Michael Kessler, Curtis Henn, Ryan Murray

**Affiliations:** Medstar Georgetown University Hospital, Washington, DC, USA

**Keywords:** carpal tunnel release, collegiate athletes, median nerve compression, open reduction internal fixation, return to sport, trans-scaphoid perilunate fracture-dislocation

## Abstract

**Background:**

Trans-scaphoid perilunate fracture-dislocation (TSPLFD) is an uncommon but high-risk injury in athletes, and acute median nerve symptoms can influence surgical timing and approach.

**Case presentation:**

We report two 20-year-old collegiate football players who sustained TSPLFD during play. In Case 1, closed reduction in the emergency department restored alignment, but the patient reported mild paresthesias in the median nerve distribution. He underwent open reduction and internal fixation (ORIF) of the scaphoid with lunotriquetral stabilization and carpal tunnel release. In Case 2, closed reduction was unsatisfactory, and sensory symptoms persisted, prompting emergent ORIF with carpal tunnel release. Both athletes returned to Division I collegiate football without recurrent neurologic symptoms.

**Clinical discussion:**

These cases highlight (1) the importance of prompt recognition and advanced imaging to define the injury pattern, (2) the rationale for emergent surgical management when reduction is inadequate or neurologic symptoms persist, and (3) the need to document objective postoperative outcomes in high-demand athletes. We also discuss indications for urgent carpal tunnel release in the setting of perilunate injuries and relate decision-making to the available evidence.

**Conclusion:**

TSPLFD in competitive athletes can allow return to sport when treated with timely reduction, stable fixation, and a low threshold for carpal tunnel release when acute median nerve symptoms persist or worsen. However, short-term follow-up cannot exclude long-term risks such as post-traumatic arthritis and avascular necrosis, and athletes should be counseled accordingly.

## Introduction

Perilunate dislocations occur after an axial load to the thenar eminence with the wrist forced into extension. When the injury includes a scaphoid fracture, it is classified as a trans-scaphoid perilunate fracture-dislocation (TSPLFD), which accounts for approximately 60% of perilunate injuries^[^1,2^]^. Patients present with wrist swelling and anatomic snuffbox tenderness. The Mayfield classification describes progressive perilunate instability across four stages, from scapholunate diastasis to volar lunate dislocation[3]. Radiographs may show disruption of Gilula’s arcs; on the lateral view, the “spilled teacup sign” is characteristic of Mayfield stage 4[4].

HIGHLIGHTS

- Rare trans-scaphoid perilunate fracture-dislocations in collegiate athletes.
- Median nerve compression requiring urgent carpal tunnel release.
- Open reduction with screw and K-wire fixation restored wrist alignment.
- Athletes successfully returned to competitive sports after surgery.

In athletes, these injuries occur in-season and compress rehabilitation timelines. Acute median nerve symptoms are reported in approximately 24–45% of perilunate injuries, adding urgency to management and potentially necessitating decompression through a combined dorsal-volar approach^[^4,5^]^. Clinicians frequently miss perilunate injuries at initial presentation; delayed diagnosis increases the risk of avascular necrosis and chronic carpal instability[4]. We report two collegiate football players with TSPLFD and acute median nerve symptoms treated with open reduction and internal fixation (ORIF) and carpal tunnel release, emphasizing decision-making around urgent decompression, operative stabilization, and return-to-sport counseling. Written informed consent was obtained. This report follows SCARE 2025 guidelines[6].

## Case presentation

### Case 1

**Figure 1.**
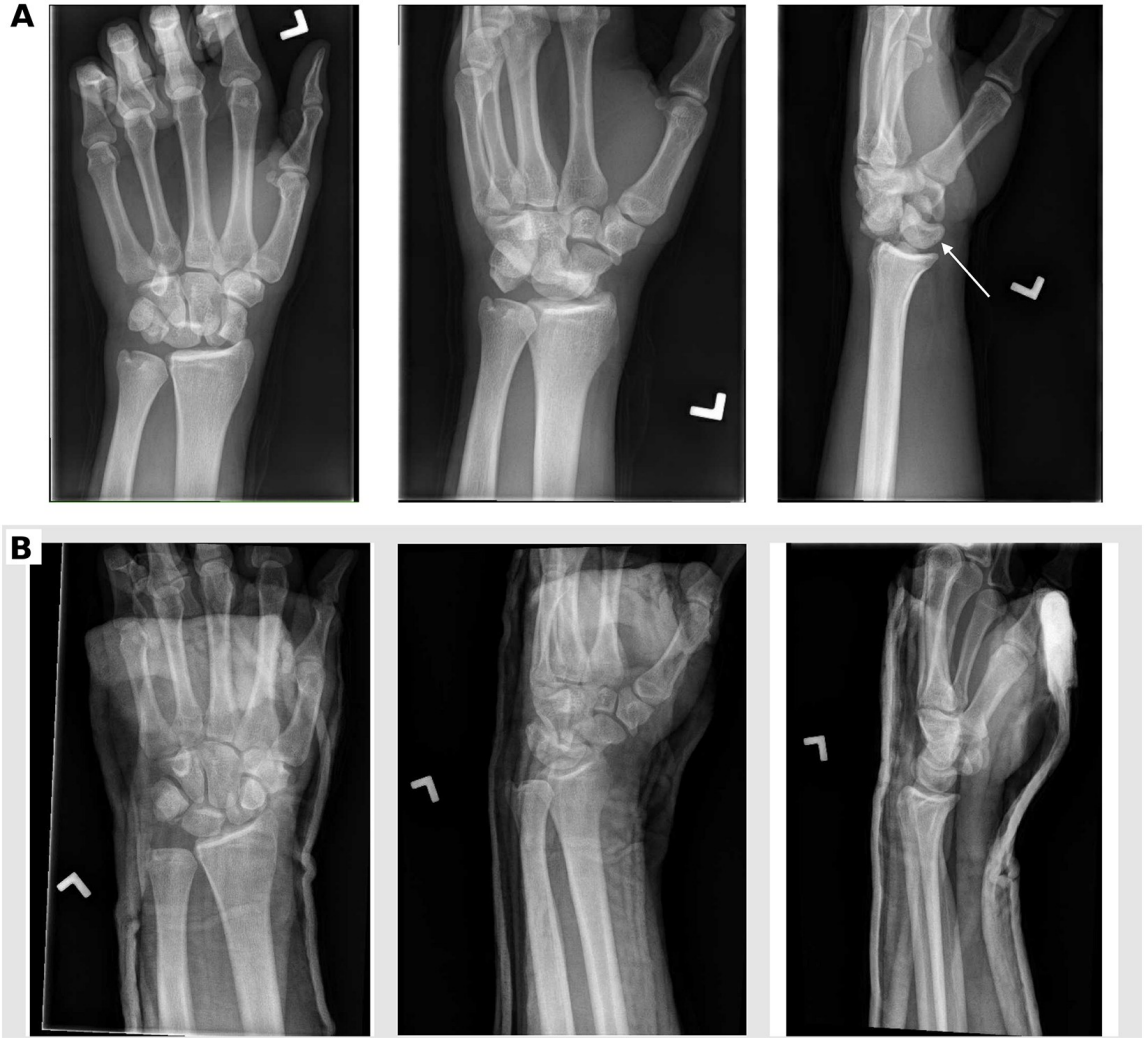
(A) (Case 1, initial injury – pre-reduction): Three-view radiographs of the left wrist demonstrate a trans-scaphoid perilunate fracture-dislocation, with dorsal displacement of the capitate/carpus relative to the lunate on the lateral view, consistent with a perilunate injury pattern. (B) (Case 1, post-reduction – emergency department): Three-view radiographs of the left wrist demonstrate improved carpal alignment after closed reduction. Residual malalignment is present, with radial translation/subluxation of the capitate relative to the lunate, as well as an associated scaphoid fracture and triquetral fracture/avulsion.

A 20-year-old male collegiate football player landed on his left wrist during a game and sustained a TSPLFD with a triquetral avulsion. He reported mild paresthesias in the volar thumb, index, and long fingers, with intact median motor function. Three-view radiographs demonstrated the injury (Fig. 1A). In the emergency department, closed reduction under conscious sedation was performed and confirmed with fluoroscopy; a sugar-tong splint was applied (Fig. 1B). Post-reduction computed tomography (CT) confirmed nondisplaced fractures of the scaphoid and triquetrum.

Four days later, he underwent operative management under general anesthesia with a regional block. Through a dorsal approach ulnar to Lister’s tubercle, the surgeon performed scaphoid ORIF with two Synthes cannulated headless compression screws (2.5-mm and 2.0-mm) placed proximal to distal, confirmed an intact scapholunate ligament, and placed a 0.045-inch K-wire from the scaphoid to the capitate. A torn lunotriquetral ligament was repaired with a 0.045-inch K-wire and an Arthrex knotless suture anchor. The surgeon then performed open carpal tunnel release through a volar longitudinal incision, released the transverse carpal ligament, visualized the median nerve, and evacuated old hematoma from the carpal tunnel. Median nerve symptoms resolved postoperatively.

**Figure 2.**
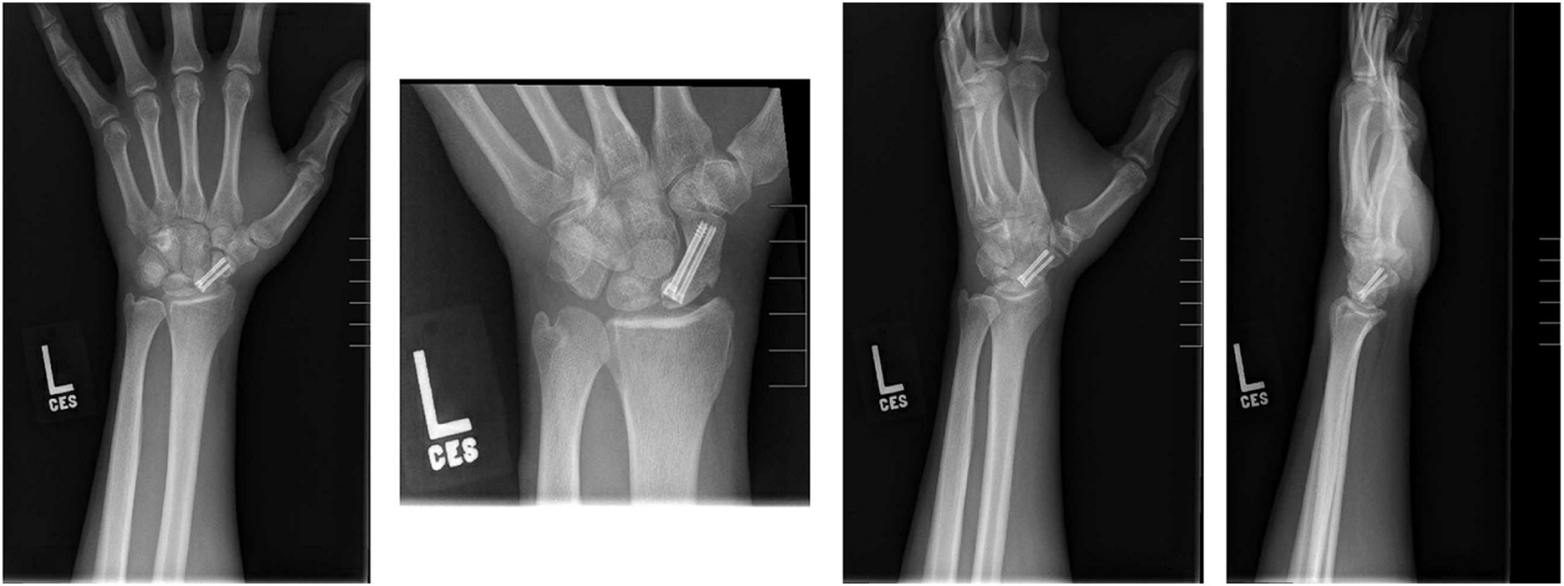
(Case 1, postoperative month 6): Four-view radiographs of the left wrist demonstrating the interval removal of K-wires, two retained scaphoid screws, and maintained carpal alignment without hardware complications.

At 11 weeks, K-wires were removed, and occupational therapy-directed range-of-motion exercises began. At 7 months, active wrist range of motion (ROM) on the injured side measured 50° extension and 35° flexion (versus 70°/70° contralateral); passive ROM measured 50° extension and 40° flexion, limited by adhesions. He returned to collegiate football at 6 months postoperatively. Follow-up radiographs demonstrated interval K-wire removal, two retained scaphoid screws, and maintained carpal alignment (Fig. 2).

#### Timeline (Case 1)

- **Day 0 (Injury**): Left TSPLFD with triquetral avulsion during a game. Mild median-distribution paresthesias, motor intact. Underwent closed reduction under conscious sedation with fluoroscopic confirmation and was placed in a sugar-tong splint.
- **Day 0–1:** Post-reduction CT: nondisplaced scaphoid and triquetral fractures.
- **Day 4:** Left TSPLFD with triquetral avulsion during a game. Mild median-distribution paresthesias, motor intact. Underwent closed reduction under conscious sedation with fluoroscopic confirmation and was placed in a sugar-tong splint.
- **Postoperative week 1:** Follow-up radiographs were obtained; transitioned to a short-arm thumb spica cast.
- **Postoperative week 11**: Follow-up radiographs were obtained; transitioned to a short-arm thumb spica cast.
- **Postoperative month 6:** Returned to collegiate football.
- **Postoperative month 7:** Occupational therapy exam showed wrist active ROM of 50° extension and 35° flexion on the injured side versus 70°/70° contralaterally; radial and ulnar deviation were both 15°. Passive ROM was 50° extension and 40° flexion, limited by adhesions/scarring.

### Case 2

**Figure 3.**
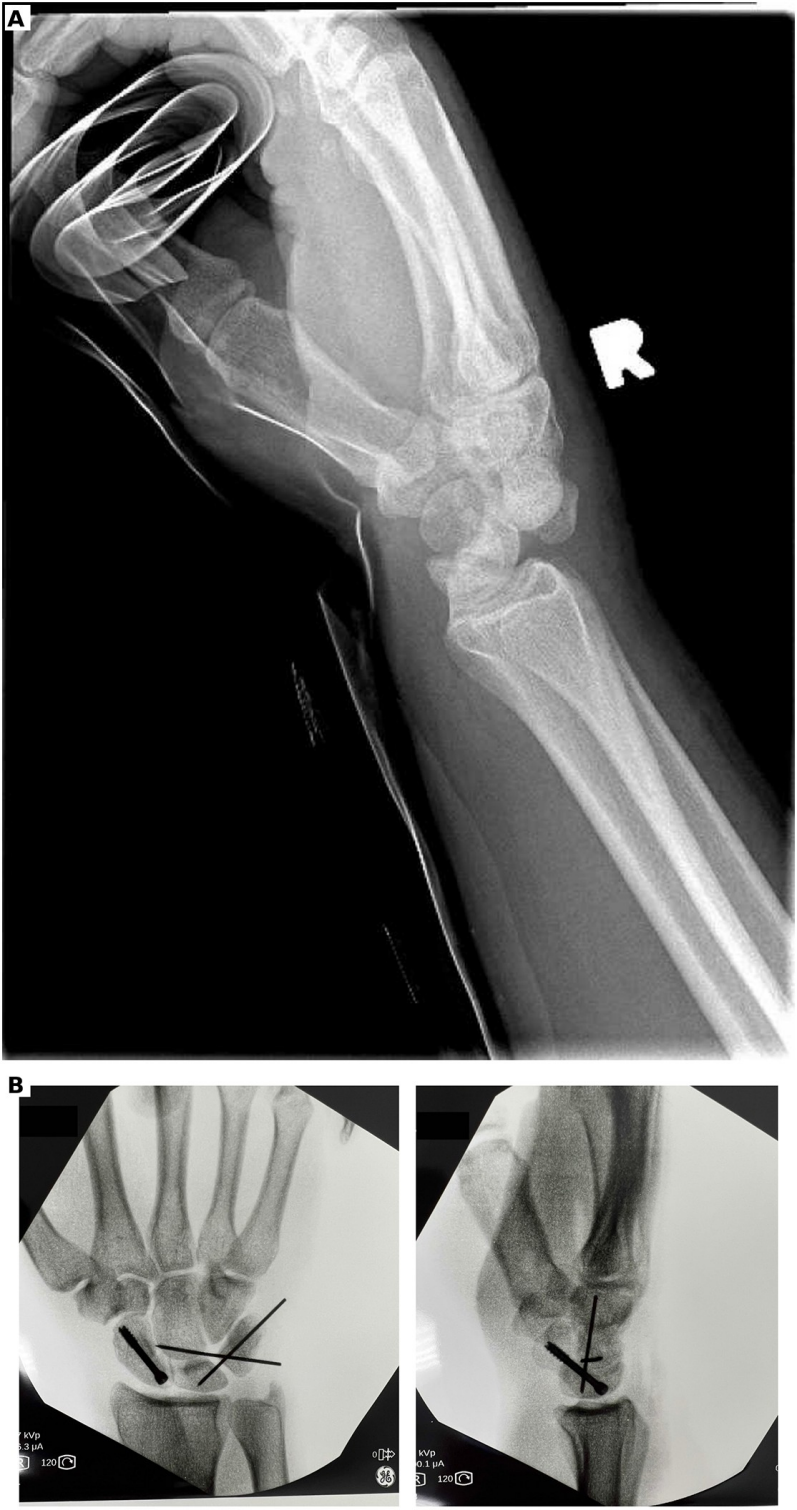
(A) (Case 2, initial injury – pre-reduction): Lateral radiograph of the right wrist demonstrating a perilunate fracture-dislocation with dorsal displacement of the capitate/carpus relative to the lunate and an associated scaphoid fracture at the junction of the proximal and middle thirds. (B) (Case 2, immediate postoperative/intraoperative fluoroscopy): Anterior-posterior (AP) and lateral fluoroscopic images of the right wrist demonstrating anatomic reduction of the scaphoid fracture with headless compression screw fixation, restored carpal alignment, and temporary K-wire stabilization across the lunotriquetral and midcarpal joints.

A 20-year-old right-hand-dominant male collegiate football player landed on his right hand with the wrist in extension during a game and sustained a TSPLFD. He reported decreased sensation in the median nerve distribution. A lateral radiograph confirmed a perilunate fracture-dislocation with an associated scaphoid fracture at the junction of the proximal and middle thirds (Fig. 3A). An attempted closed reduction under conscious sedation was unsatisfactory, and sensory symptoms persisted, prompting emergent operative management.

Under general anesthesia, the surgeon first performed an open carpal tunnel release through a standard volar longitudinal incision. Hematoma and bright red blood were evacuated from the carpal tunnel; the transverse carpal ligament was completely released proximally and distally. A dorsal approach was then performed through a longitudinal incision just ulnar to Lister’s tubercle. After posterior interosseous nerve neurectomy and capsulotomy, the surgeon achieved anatomic reduction of the displaced scaphoid fracture and fixed it with a Synthes 3.0-mm partially threaded headless compression screw (24-mm). Due to residual lunotriquetral and midcarpal instability, the surgeon placed 0.045-inch K-wires across the triquetrum-to-lunate and triquetrum-to-capitate joints. Final fluoroscopy confirmed anatomic reduction, hardware position, and carpal alignment (Fig. 3B). Total tourniquet time was 2 hours and 7 minutes.

On postoperative day 1, sensation was intact in all nerve distributions, and motor function was fully preserved; the patient was discharged with plans to follow up with his home orthopedic surgeon. Because follow-up occurred outside our institution, standardized objective outcome measures (wrist ROM, grip strength, disabilities of the arm, shoulder, and hand/patient-rated wrist evaluation (DASH/PRWE) were not obtainable. At 1 year postoperatively, the patient returned to Division I collegiate football and remained symptom-free.

#### Timeline (Case 2)

- **Day 0 (Injury**): Sustained a right TSPLFD during a collegiate football game after landing on an extended wrist. Reported decreased sensation in the median nerve distribution.
- **Day 0 (Emergency department**): Attempted closed reduction under conscious sedation was unsuccessful, with persistent median nerve sensory symptoms.
- **Day 0 (Operative management**): Underwent emergent ORIF under general anesthesia: open carpal tunnel release with hematoma evacuation, dorsal approach with PIN excision, scaphoid ORIF with a Synthes 3.0-mm partially threaded headless screw (24 mm), and K-wire stabilization of the triquetrum-lunate and triquetrum-capitate. Final fluoroscopy confirmed anatomic reduction, hardware position, and carpal alignment. Tourniquet time was 2:07. The patient was placed in a volar splint.
- **Postoperative day 1:** Neurovascular exam was intact, with preserved digital ROM, intact sensation, and motor function, 2+ radial pulse, and well-perfused digits. Discharged home with follow-up planned with his home orthopedic physician.
- **Postoperative month 12**: Returned to Division I collegiate football and remained symptom-free. Standardized objective outcomes were unavailable because follow-up occurred outside Medstar Georgetown University Hospital.

## Discussion

In a review of 10 professional football players with perilunate dislocations treated operatively, none were forced to retire, and 9 returned to play by the following season[7]. However, longer-term series report post-traumatic arthritis in up to 56% of TSPLFD injuries, and delayed diagnosis increases the risk of avascular necrosis and chronic instability[8]. Our cases illustrate the spectrum of TSPLFD: Case 1 reduced acceptably in the emergency department and allowed semi-urgent fixation, while Case 2 remained grossly unstable despite reduction under anesthesia, necessitating emergent ORIF to protect cartilage and neurovascular structures^[^1,9^]^.

Median nerve symptoms occur in approximately 24–45% of perilunate injuries, often resulting from increased carpal tunnel pressure due to volar carpal translation, fracture hematoma, and acute swelling^[^4,10^]^. Persistent or worsening symptoms, objective sensory loss, or emerging motor weakness after attempted reduction should prompt urgent decompression^[^11,12^]^. Opinion varies on whether to decompress routinely at the index operation versus selectively, but we applied a low threshold: symptoms persisted in Case 2 and remained present with concern for hematoma-related compression in Case 1; hematoma was identified in the carpal tunnel in both cases, supporting index decompression[13].

Anatomic scaphoid reduction and stable headless compression screw fixation supported earlier motion in both patients. Intraoperative stability testing directed tailored carpal stabilization: in Case 1, lunotriquetral repair with a knotless suture anchor was performed because the tear appeared repairable; in Case 2, persistent midcarpal instability required additional K-wire stabilization[14]. The dorsal approach facilitated reduction and intercarpal assessment, while the volar incision enabled carpal tunnel release – a combined approach consistent with published operative descriptions when median nerve decompression is required[4].

Both athletes returned to Division I collegiate football (Case 1 at 6 months, Case 2 at 12 months), consistent with published athlete series[7]. However, Case 1 demonstrated persistent motion deficits at 7 months, and Case 2 lacked longitudinal objective data. These findings reinforce that return to sport may precede clinically apparent complications; athletes should be counseled that short-term recovery does not eliminate long-term risks of stiffness and arthrosis[15]. This report is limited by its small size, heterogeneous follow-up, and absence of standardized patient-reported outcomes and grip strength testing.

Despite these limitations, our cases support three practical points: (1) recognize perilunate injury patterns early and use CT when radiographs do not fully define the injury; (2) prioritize anatomic scaphoid reduction and tailor intercarpal stabilization based on dynamic intraoperative stability; and (3) maintain a low threshold for carpal tunnel release when median nerve symptoms persist after reduction or worsen during serial examinations.

## Conclusion

Early recognition and definitive management of TSPLFD are essential, particularly when patients report median nerve symptoms. Because pain can obscure the motor exam, clinicians should rely on serial sensory findings and a careful history to identify evolving acute carpal tunnel syndrome. We recommend a low threshold for carpal tunnel release when symptoms persist after reduction, worsen over time, or include objective sensory loss or motor weakness. With timely reduction, stable fixation, and an organized rehabilitation plan, competitive athletes may return to play; however, clinicians should counsel patients that short-term recovery does not eliminate the long-term risk of stiffness and post-traumatic arthritis.

## Data Availability

Not applicable.
